# Gut microbiota and bile acids profiles study of ulcerative colitis and Crohn’s disease patients

**DOI:** 10.3389/fmicb.2026.1782415

**Published:** 2026-04-07

**Authors:** Zhejiong Wang, Jiayi Shi, Yan Yu, Linchao Zhu, Lichen Zhu, Wenjuan Tuo, Juntao Ni, Yi Wu, Shengliang Qiu, Ying Yu

**Affiliations:** 1 Department of Laboratory Medicine, The First Affiliated Hospital of Zhejiang Chinese Medical University (Zhejiang Provincial Hospital of Chinese Medicine), Hangzhou, China; 2 Haining Hospital of Traditional Chinese Medicine, Jiaxing, China; 3 Hangzhou Health-Bank Medical Laboratory Co., Ltd., Hangzhou, China; 4 Faculty of Medicine, The First School of Clinical Medicine, Yangzhou University, Yangzhou, China; 5 Artificial Intelligence and Big Data Center (Information Center), The First Affiliated Hospital of Zhejiang Chinese Medical University (Zhejiang Provincial Hospital of Chinese Medicine), Hangzhou, China; 6 The Second Affiliated Hospital of Zhejiang University, Hangzhou, China; 7 The First School of Clinical Medicine, Zhejiang Chinese Medical University, Hangzhou, Zhejiang, China; 8 The First Affiliated Hospital of Zhejiang Chinese Medical University (Zhejiang Provincial Hospital of Chinese Medicine), Hangzhou, China

**Keywords:** bile acid profiles, Crohn’s disease, *Escherichia-Shigella*, gut microbiota, *Ruminococcus_gnavus_group*, short-chain fatty acid-producing bacteria, ulcerative colitis

## Abstract

**Introduction:**

The global incidence of inflammatory bowel disease (IBD) is increasing, with dysbiosis of the gut microbiota identified as a significant factor contributing to chronic intestinal inflammation. This study aims to elucidate the gut microbiota and serum bile acid profiles in patients with ulcerative colitis (UC) and Crohn’s disease (CD), and to explore the relationship between bacterial taxa and bile acids.

**Methods:**

We recruited 31 UC patients, 41 CD patients, and 32 healthy controls from The First Affiliated Hospital of Zhejiang Chinese Medical University. Gut microbiota composition was analyzed by 16S rDNA sequencing of stool samples, and serum bile acid profiles were quantified using LC–MS/MS. Alpha and beta diversity were assessed, and differential abundance of bacterial genera was evaluated using non-parametric tests with false discovery rate (FDR) correction. Correlations between differentially abundant genera and bile acid levels were examined by Spearman’s rank correlation.

**Results:**

Both UC and CD groups exhibited significant alterations in alpha and beta diversity compared to controls. At the genus level, pro-inflammatory taxa, including *Escherichia-Shigella* and *Ruminococcus_gnavus_group,* were enriched, while short-chain fatty acid (SCFA)-producing genera such as *Coprococcus* were depleted. Notably, SCFA-producing *Romboutsia* and *Butyricicoccus* were exclusively depleted in CD. A consistent depletion of secondary bile acids—lithocholic acid (LCA), glycolithocholic acid (GLCA), deoxycholic acid (DCA), taurodeoxycholic acid (TDCA), and glycodeoxycholic acid (GDCA)—was observed in both patient groups. Correlation analysis revealed positive associations between several *Lachnospiraceae* genera and DCA/LCA levels.

**Discussion:**

This study confirms gut microbiota dysbiosis and altered bile acid profiles in UC and CD, with CD displaying a more pronounced disruption of SCFA-producing bacteria. The observed depletion of *Lachnospiraceae* and secondary bile acids suggests a link between microbial metabolic dysfunction and IBD pathogenesis. Although *Lachnospiraceae* may represent a candidate for next-generation probiotic development, further mechanistic and clinical studies are required to validate its therapeutic potential.

## 1 Introduction

Inflammatory bowel disease (IBD), primarily comprising ulcerative colitis (UC) and Crohn’s disease (CD), is a group of chronic, relapsing gastrointestinal disorders driven by immune dysregulation (Kaser et al., 2010). Common symptoms—recurrent abdominal pain, diarrhea, bloody stools, and weight loss—often persist or recur, substantially impairing quality of life (Kaser et al., 2010). The global incidence and prevalence of IBD have risen steadily in recent decades, a trend linked to modernization, dietary shifts, and environmental changes; currently, over 10 million individuals are affected worldwide (Kaplan and Windsor, 2021). This increase is particularly evident in China, where improved diagnostic capacity and Westernized lifestyles have contributed to a growing disease burden. Epidemiological estimates indicate a UC prevalence of approximately 11.6 per 100,000 and CD prevalence of 2.29 per 100,000, with projections suggesting that the total IBD population in China will exceed 1.5 million by 2025 (Ng et al., 2017; Loftus J., 2004).

Although the exact pathogenesis of IBD remains incompletely understood, it is widely accepted as a multifactorial process involving genetic susceptibility, environmental triggers, gut microbiota dysbiosis, intestinal barrier dysfunction, and aberrant immune responses (Ma et al., 2025). Among these, gut microbiota dysbiosis has emerged as a central driver of chronic intestinal inflammation (Asquith and Powrie, 2010). IBD-associated dysbiosis is characterized by reduced microbial diversity, diminished stability, and altered abundances of specific taxa (Sartor, 2008). A consistent hallmark is the depletion of short-chain fatty acid (SCFA)-producing bacteria (e.g., *Bifidobacterium*, *Odoribacter*, *Butyricicoccus*, *Roseburia*, *Lachnospira*) alongside enrichment of pro-inflammatory taxa such as *Escherichia-Shigella* and *Ruminococcus gnavus* (Ahmadi et al., 2025; Vallejos et al., 2025; Gomes et al., 2024; Zhou et al., 2022; Eeckhaut et al., 2013; Kou et al., 2025; Scaldaferri et al., 2025; Kim et al., 2024; Wang et al., 2024; Hall et al., 2017; Volkmann, 2023).

A key functional consequence of this ecological disturbance is the disruption of bile acid metabolism. Secondary bile acids (SBAs)—including deoxycholic acid (DCA) and lithocholic acid (LCA)—are generated from primary bile acids exclusively through the enzymes produced by gut bacteria and are now recognized as important immunomodulators (Fu et al., 2025; Jiao et al., 2018). Numerous studies have reported decreased levels of SBAs and their conjugates in IBD patients (Cai et al., 2022; Lloyd-Price et al., 2019; Franzosa et al., 2019; Sinha et al., 2020; Xu et al., 2024), while certain SBA metabolites (e.g., 3-oxoLCA, isoLCA, TDCA) exert protective, anti-inflammatory effects (Zou et al., 2023; Paik et al., 2022). Thus, SBAs represent a critical functional link between gut microbiota and host immune homeostasis in IBD.

Despite these advances, comparative analyses of microbial and metabolic disturbances between UC and CD—the two clinically distinct IBD subtypes—remain limited (Yang et al., 2019). Moreover, systematic exploration of correlations between altered bacterial genera and serum bile acid profiles in IBD patients is needed to bridge taxonomic observations with functional metabolic outcomes.

Therefore, this study aimed to conduct a parallel analysis of the gut microbiota and serum bile acid profiles in UC and CD patients. We sought not only to compare dysbiotic patterns between subtypes but also to identify specific correlations between differential bacterial genera and bile acid levels. We hypothesized that such an integrated approach would reveal distinct, subtype-specific microbial-metabolic axes. These findings are expected to deepen mechanistic understanding of IBD heterogeneity and provide a rational basis for developing targeted next-generation probiotic or postbiotic therapies.

## 2 Materials and methods

### 2.1 Participants and samples

A total of 104 participants were recruited from The First Affiliated Hospital of Zhejiang Chinese Medical University (Zhejiang Provincial Hospital of Chinese Medicine) based on standard endoscopic, radiographic, and histologic criteria, including 31 UC patients, 41 CD patients, and 32 age- and sex-matched health check-up population without IBD as healthy controls. Fecal samples (≥2 g) were collected from the central portion using sterile spoons, immediately transferred to cryotubes containing protective reagents, and stored at −80 °C. Fasting venous blood samples (5 mL) were collected in the morning, centrifuged at 3000 rpm for 10 min within 2 h of collection, and the serum supernatant was stored at −80 °C.

### 2.2 16S rDNA sequencing

Total bacterial genomic DNA was extracted from fecal samples using the PowerMax Stool/Soil DNA Isolation Kit (MoBio Laboratories, Carlsbad, CA, United States) according to the manufacturer’s instructions. The extracted DNA was stored at −20 °C until further analysis. DNA concentration and purity were assessed with a NanoDrop ND-1000 spectrophotometer (Thermo Fisher Scientific, Waltham, MA, United States), and integrity was evaluated by agarose gel electrophoresis.

The V4 region of the bacterial 16S rRNA genes was amplified by PCR using the forward primer 515F (5′-GTGCCAGCMGCCGCGGTAA-3′) and the reverse primer 806R (5′-GGACTACHVGGGTWTCTAAT-3′). Sample-specific 6-bp barcodes were incorporated using TrueSeq adapters to facilitate multiplex sequencing. Each 50 μL PCR reaction contained 25 μL Phusion High-Fidelity PCR Master Mix, 3 μL (10 μM) each of forward and reverse primers, 10 μL DNA template, 3 μL DMSO, and 6 μL ddH₂O. Thermal cycling conditions were: initial denaturation at 98 °C for 30 s; 25 cycles of 98 °C for 15 s, 58 °C for 15 s, 72 °C for 15 s; and final extension at 72 °C for 1 min. Amplicons were purified using Agencourt AMPure XP Beads (Beckman Coulter, Indianapolis, IN, United States), quantified with the PicoGreen dsDNA Assay Kit (Invitrogen, Carlsbad, CA, United States), pooled in equimolar amounts, and subjected to paired-end sequencing (2 × 150 bp) on an Illumina NovaSeq 6,000 platform.

Raw sequencing data were quality-filtered using FASTP v0.20.1. Operational taxonomic units (OTUs) were clustered at 97% similarity using Vsearch v2.15.0. Alpha diversity indices (Chao1, Simpson, Shannon) and beta diversity (Weighted UniFrac principal coordinate analysis, PCoA) were calculated using QIIME2 v2022.6. Linear discriminant analysis effect size (LEfSe) was performed with an LDA score threshold >3.0 and *p* < 0.05 to identify differentially abundant taxa.

Based on the random forest (RF) model, UC and CD specific microbial markers were used to construct a classification model with stratified 10-fold cross-validation.

### 2.3 Bile acid quantification by LC–MS/MS

Bile acids engage in a bidirectional relationship with the gut microbiota, being metabolized by it while also shaping its composition. Given the established link between bile acids and inflammatory bowel disease (IBD) (Cai et al., 2022; Guo et al., 2022), we quantified serum bile acid profiles using LC–MS/MS.

Serum samples (100 µL) were mixed with 300 µL of methanol (containing the internal standard solution), vortexed for 5 min, and centrifuged at 12,000 rpm for 5 min at 4 °C. Then 150 μL of the supernatant was transferred and mixed with 150 μL of pure water. After vortexing and cntrifugation, the supernatant was collected for injection.

Chromatographic separation was performed on a LC-MS/MS system coupled to a TSQ mass spectrometer (Thermo, USA) using a Phenomenex Column (100 × 3 mm, 2.6 µm). Mobile phase A was water (containing 10 mmol/L ammonium acetate), mobile phase B was acetonitrile. The gradient elution program at 0.4 mL/min and 40 °C was: 0-5 min, 35-40% B; 5-5.5 min, 40-100% B, and held for 0.5 min; 6-6.5 min, 100-35% B. Injection volume was 15 µL. An Electrospray Ionization (ESI) source was operated in negative ion mode for ion generation. Ion source properties were as follows: Negative Ion was 3000 V; Ion Transfer Tube Temp was 300 °C; Vaporizer Temp was 350 °C. Quantification of the bile acid profile was performed using multiple reaction monitoring (MRM) mode.

The calibration curve was constructed by plotting the linear concentrations on the x-axis against the ratio of the response signal of the reference material to that of the corresponding internal standard on the y-axis. The concentrations of individual bile acid components in the samples were determined using the regression equation derived from the calibration curve.

### 2.4 Heatmap analysis

Spearman’s rank correlation coefficients between differentially abundant bacterial genera and bile acid concentrations (or clinical parameters) were computed and visualized as heatmaps using the “pheatmap” package in R.

### 2.5 Statistical analysis

In microbiome analysis, due to the simultaneous comparison of numerous microbial features, we applied the Benjamini–Hochberg method to control for the risk of false positives from multiple testing, performing false discovery rate (FDR) correction on all *p* values. The adjusted FDR values were subsequently used as the criterion for determining statistical significance. Given the non-normal distribution of the gut microbiota data, non-parametric tests (Wilcoxon test) were used for between-group comparisons. All statistical analyses of gut microbiota were performed using the stats package in R. Normality of bile acid data was assessed with the Shapiro–Wilk test in R version 4.3.3; since the resulting *p*-values were <0.1, subsequent comparisons of bile acids were conducted using the Mann–Whitney test in GraphPad Prism 8.0.2.

## 3 Results

Stool and serum samples were collected from 32 healthy controls and 72 patients with inflammatory bowel disease (IBD), comprising 31 ulcerative colitis (UC) and 41 Crohn’s disease (CD) cases. Gut microbiota profiling was performed using 16S rRNA sequencing of stool samples, while serum bile acid concentrations were quantified by liquid chromatography–tandem mass spectrometry (LC–MS/MS).

### 3.1 Alpha diversity and beta diversity of the gut microbiota

**Figure 1:**
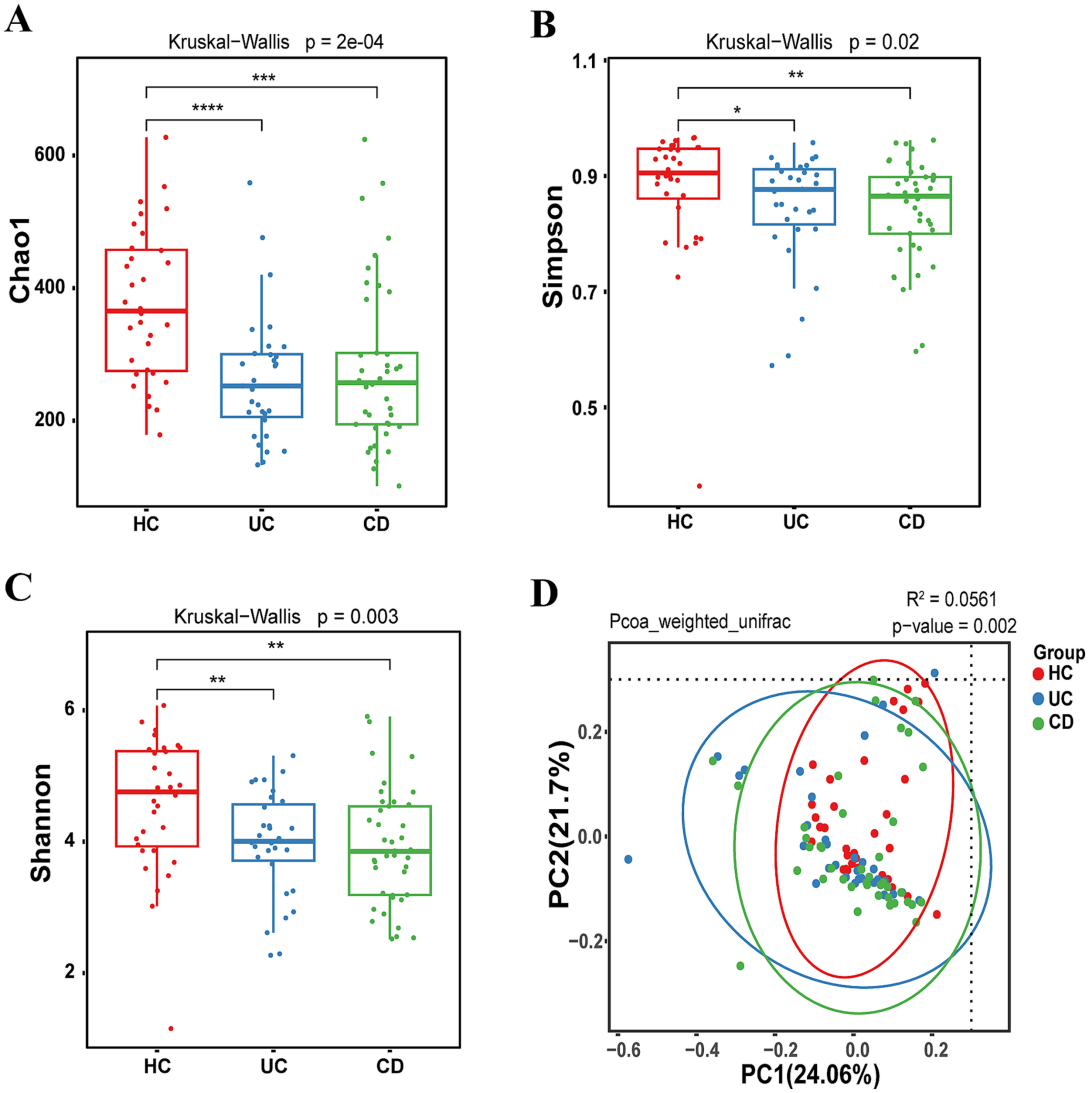
Alpha diversity and beta diversity of IBD patients and healthy control. **(A)** Chao1 index. **(B)** Simpson index. **(C)** Shannon index. **(D)** PCoA figure. *p < 0.05, **p < 0.01, ***p < 0.001.

Alpha diversity, which measures the richness and diversity of the gut microbiota community, was assessed using several indices. Notable differences were observed in the Chao1 richness index, Simpson diversity index, and Shannon diversity index between the UC group and the control group, as well as between the CD group and the control group (Figures 1A–C).

Beta diversity, which reflects the shared diversity across different ecological distances within the microbiota, was utilized to compare differences among multiple groups (Yang et al., 2022). Weighted UniFrac principal coordinate analysis (PcoA) revealed significant differences among the three groups and highlighted variations in species classification (Figure 1D).

### 3.2 Alterations in the microbiota

**Figure 2:**
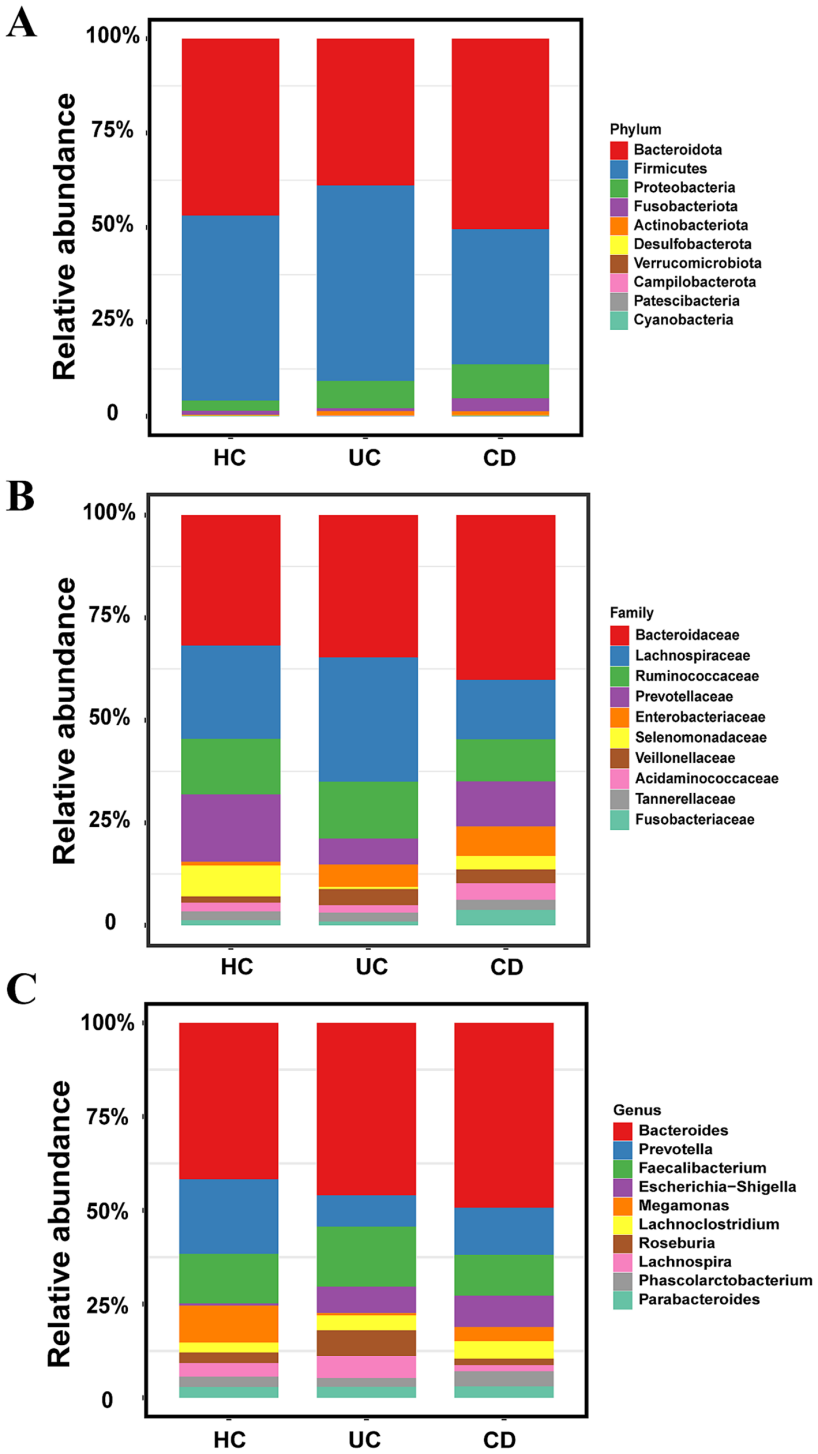
Compositional differences of taxonomic proportions (top 10): **(A)** Taxonomic proportions at the phylum level; **(B)** taxonomic proportions at the family level; **(C)** taxonomic proportions at the genus level.

Figures 2A–C illustrate distinct gut microbiota compositions between patients with inflammatory bowel disease (IBD) and healthy controls across multiple phylogenetic levels. Consistent with their clinical classifications, ulcerative colitis (UC) and Crohn’s disease (CD), the two main IBD subtypes, exhibited unique microbial profiles.

At the phylum level, the microbiota was predominantly composed of Firmicutes (35.6–51.1%) and Bacteroidota (38.3–49.7%). Comparative analysis revealed that UC patients were characterized by significant depletions in Bacteroidota (1.22-fold), Fusobacteriota (1.41-fold), Cyanobacteria (5.15-fold), Desulfobacterota (1.92-fold), and Verrucomicrobiota (5.30-fold), alongside enrichments in Proteobacteria (2.61-fold), Actinobacteriota (5.93-fold), Patescibacteria (1.45-fold), and Campilobacterota (17.56-fold). In contrast, CD patients showed a distinct pattern, marked solely by the depletion of Firmicutes (1.38-fold) but enrichments in Proteobacteria (3.27-fold), Fusobacteriota (3.10-fold), Actinobacteriota (5.47-fold), Cyanobacteria (1.61-fold), and Verrucomicrobiota (2.07-fold).

At the family level, the gut microbiota of UC patients was characterized by the depletion of Prevotellaceae (2.64-fold), Selenomonadaceae (14.05-fold), and Fusobacteriaceae (1.41-fold), and the enrichment of Lachnospiraceae (1.30-fold), Veillonellaceae (2.54-fold), and Enterobacteriaceae (5.52-fold). In contrast, CD patients showed depletion of Prevotellaceae (1.49-fold), Lachnospiraceae (5.83-fold), Ruminococcaceae (1.31-fold), and Selenomonadaceae (2.27-fold), alongside enrichment of Bacteroidaceae (1.27-fold), Acidaminococcaceae (1.91-fold), Veillonellaceae (2.22-fold), Fusobacteriaceae (3.10-fold), and Enterobacteriaceae (7.57-fold).

At the genus level, UC patients exhibited depletion of *Prevotella* (2.45-fold), *Megamonas* (14.82-fold), and *Phascolarctobacterium* (1.20-fold), and enrichment of *Lachnoclostridium* (1.47-fold), *Lachnospira* (1.56-fold), *Roseburia* (2.34-fold), and *Escherichia-Shigella* (11.76-fold). Conversely, CD patients were marked by the depletion of *Prevotella* (1.47-fold), *Lachnospira* (2.02-fold), *Roseburia* (1.52-fold), and *Megamonas* (2.41-fold), and the enrichment of *Bacteroides* (1.27-fold), *Lachnoclostridium* (1.88-fold), *Phascolarctobacterium* (1.56-fold), and *Escherichia-Shigella* (15.57-fold).

### 3.3 Diagnostic biomarkers for IBD in the gut microbiota

**Figure 3:**
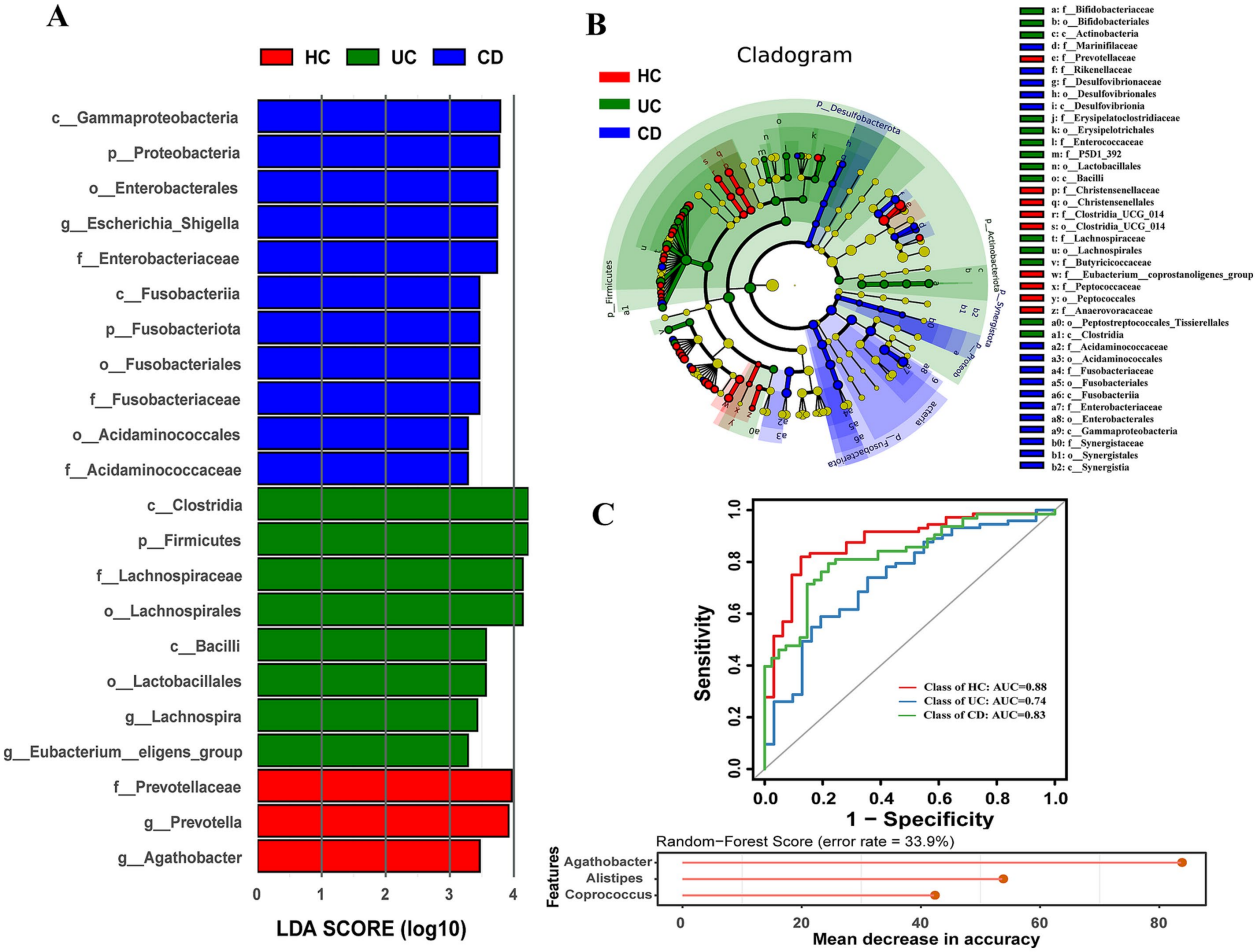
LEfSe analysis of IBD patients and healthy control. **(A)** Histogram of LDA scores (>3.0) show differentially microbial taxa. **(B)** Cladogram show branches and nodes of microbiome differences. **(C)** ROC curve and random-forest score of three microbial genus taxa.

Linear discriminant analysis effect size (LEfSe) revealed 22 differentially abundant taxa (LDA score (log10) > 3.0) among the groups, with 3, 8, and 11 taxa being enriched in the healthy controls, ulcerative colitis (UC), and Crohn’s disease (CD) groups, respectively (Figure 3A). The class Clostridia (LDA score = 4.23) was the most prominent biomarker in the UC group. In contrast, the family Prevotellaceae (LDA score = 3.97) and the class Gammaproteobacteria (LDA score = 3.79) were the most discriminating features for the healthy controls and CD groups, respectively. Phylogenetic relationships of these distinct microbial features are further visualized in the cladogram (Figure 3B).

We next evaluated the diagnostic potential of the gut microbiota using a random forest model. Initially, models based on 5 phylum, 17 family, and 20 genus taxa were constructed, showing promising AUC curves (Supplementary Figure S1). To enhance clinical applicability, a streamlined diagnostic model comprising only three genera (*Agathobacter*, *Alistipes*, and *Coprococcus*) was developed. This simplified model effectively discriminated IBD patients from healthy controls, achieving AUC values of 0.88 for healthy controls, 0.74 for UC patients, and 0.83 for CD patients (Figure 3C).

### 3.4 Differential microbial taxa at the genus level

**Figure 4:**
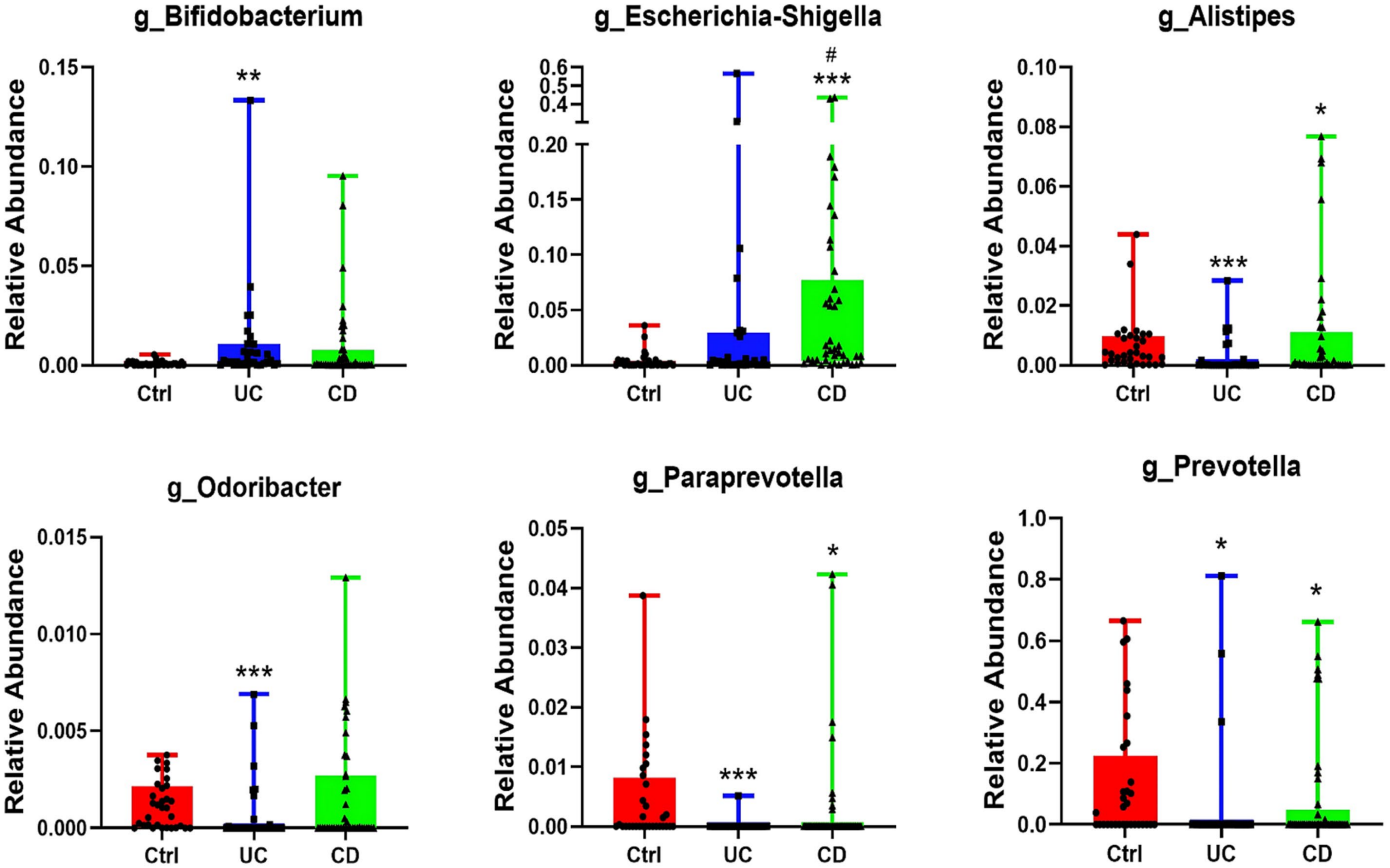
Differential genus taxa of non-*p*_Firmicutes. Six of 32 differential genus taxa were belonged to non-*p*_Firmicutes, among them, pro-inflammatory genus *Escherichia-Shigella* was significantly enriched in CD patients. Compared with HC, *p < 0.05, **p < 0.01, ***p < 0.001. Compared with UC, #*p* < 0.05.

At the genus level, we identified 32 taxa that significantly differed between IBD patients and healthy controls, with the majority classified under the phylum Firmicutes. Notably, several genera from the order Bacteroidales were consistently depleted: *Paraprevotella*, *Prevotella*, and *Alistipes* were reduced in both UC and CD patients, while *Odoribacter* was specifically depleted in UC. Conversely, the pro-inflammatory genus *Escherichia-Shigella* was uniquely and markedly enriched (~15.62-fold) in CD patients (Figure 4).

**Figure 5:**
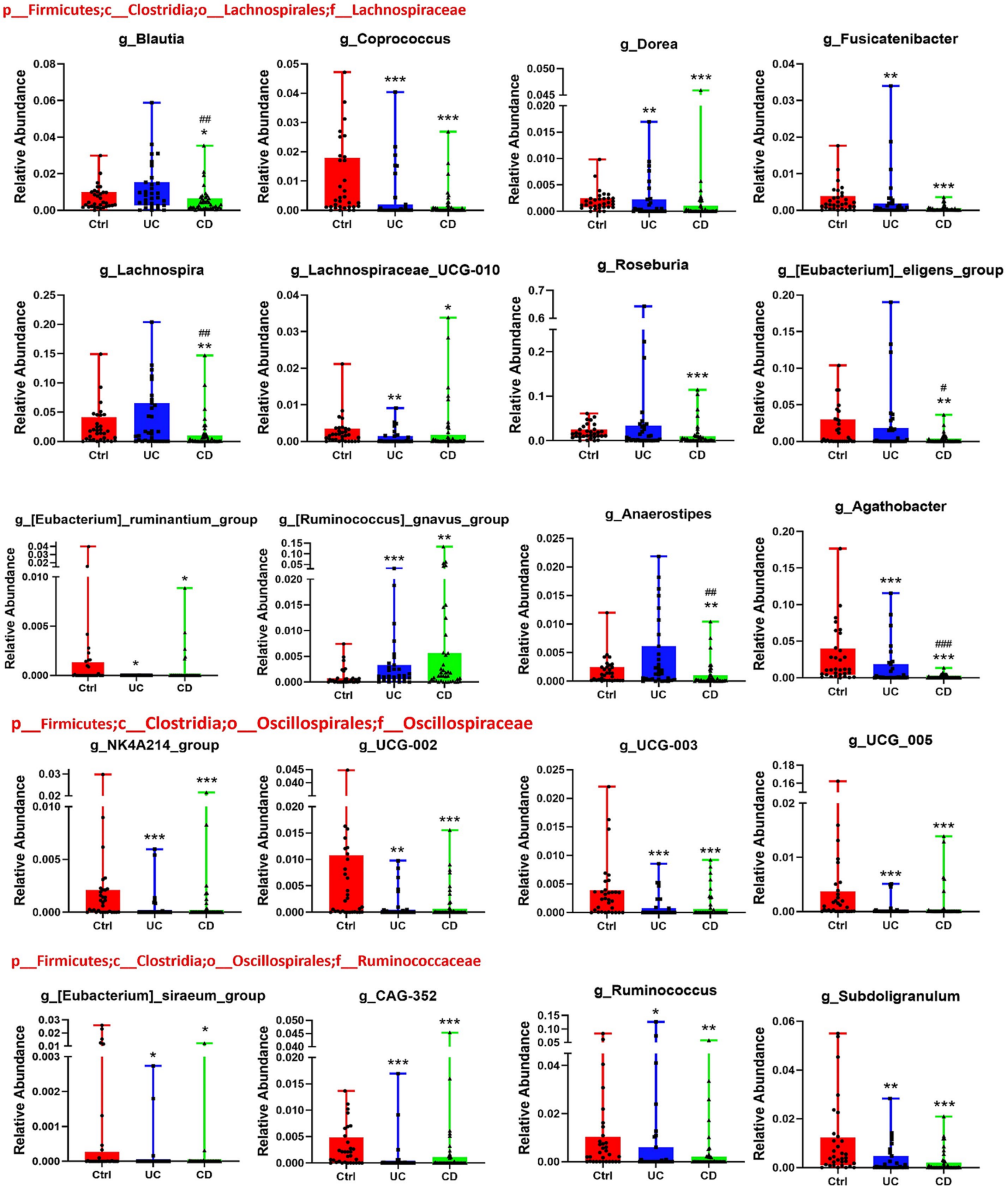
Differential genus taxa of *c_Clostridia*. 20 of 32 differential genus taxa belonged to *c_Clostridia*, including Lachnospiraceae, Oscillospiraceae, and Ruminococcaceae family. Compared with HC, *p < 0.05, **p < 0.01, ***p < 0.001. Compared with UC, #*p* < 0.05, ##*p* < 0.01, ###*p* < 0.001.

Among the 32 differentially abundant genera, the majority belonged to the class Clostridia, a principal SCFA-producing group. Within this class, all eight differential genera from Oscillospiraceae and Ruminococcaceae were depleted in both UC and CD (Figure 5). Within Lachnospiraceae, 11 of 12 differential genera were depleted in CD, whereas only 6 (*Coprococcus, Dorea, Fusicatenibacter, Lachnospiraceae_UCG-010, Agathobacter, Eubacterium ruminantium group*) were depleted in UC (Figure 5). Importantly, *Romboutsia* and *Butyricicoccus*—both SCFA producers—were depleted exclusively in CD (Supplementary Figure S2), suggesting a more profound disruption of microbial metabolic capacity in this subtype.

### 3.5 Bile acids profile of IBD patients and healthy controls

**Figure 6:**
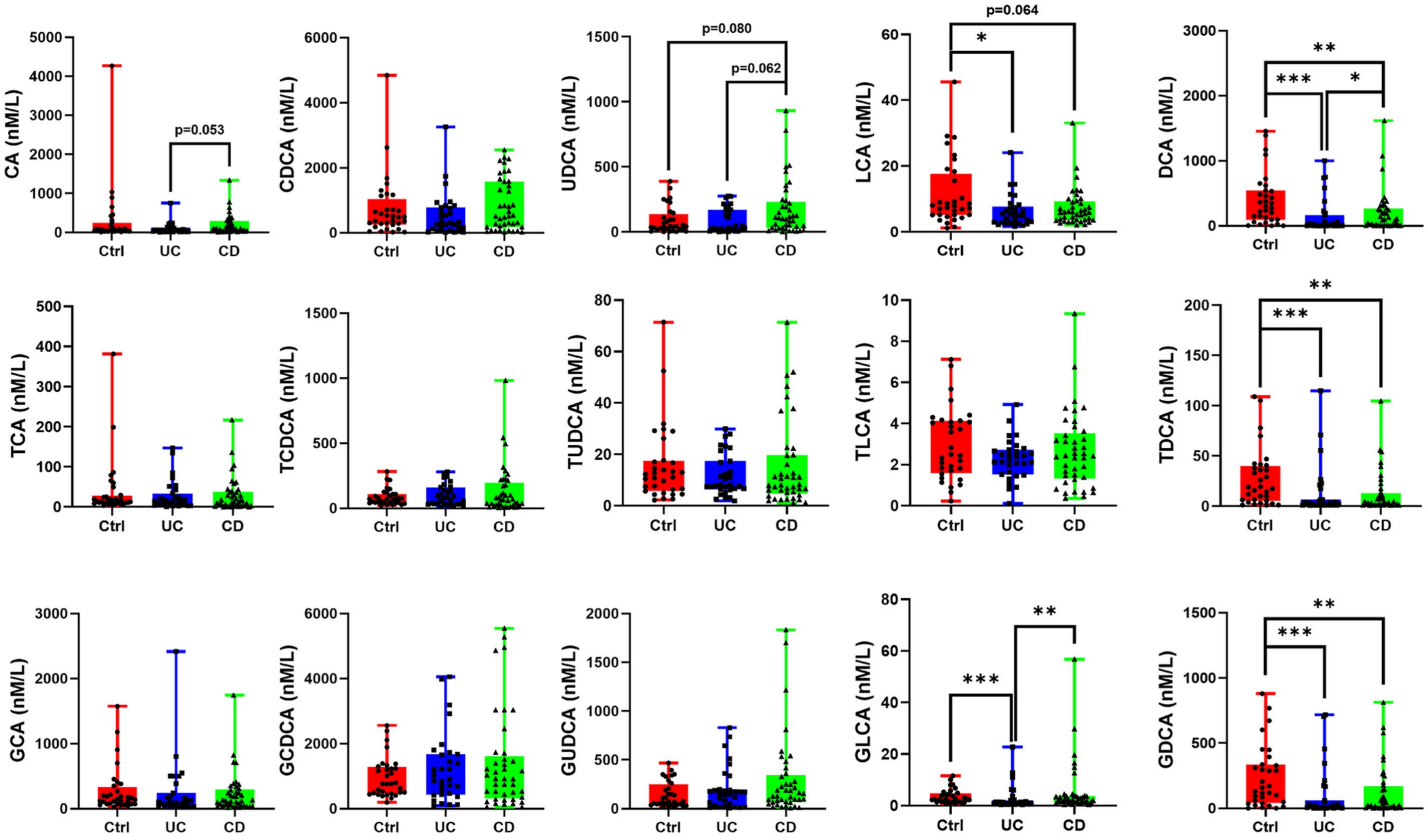
Bile acids profile of IBD patients and healthy controls. Secondary bile acids DCA, TDCA, and GDCA were depleted in both UC and CD patients, while LCA and GLCA were depleted in UC patients. *p < 0.05, **p < 0.01, ***p < 0.001.

Our analysis revealed that the levels of primary bile acids—cholic acid (CA) and chenodeoxycholic acid (CDCA)—and their conjugated forms (TCA, GCA, TCDCA, GCDCA) remained unchanged across groups. In contrast, a consistent depletion of secondary bile acids was observed in IBD. Specifically, deoxycholic acid (DCA) and its conjugates (TDCA, GDCA) were significantly reduced in both UC and CD patients. Lithocholic acid (LCA) and GLCA were significantly decreased in UC patients (Figure 6).

### 3.6 Correlation analysis between bile acids and differential genus taxa

**Figure 7:**
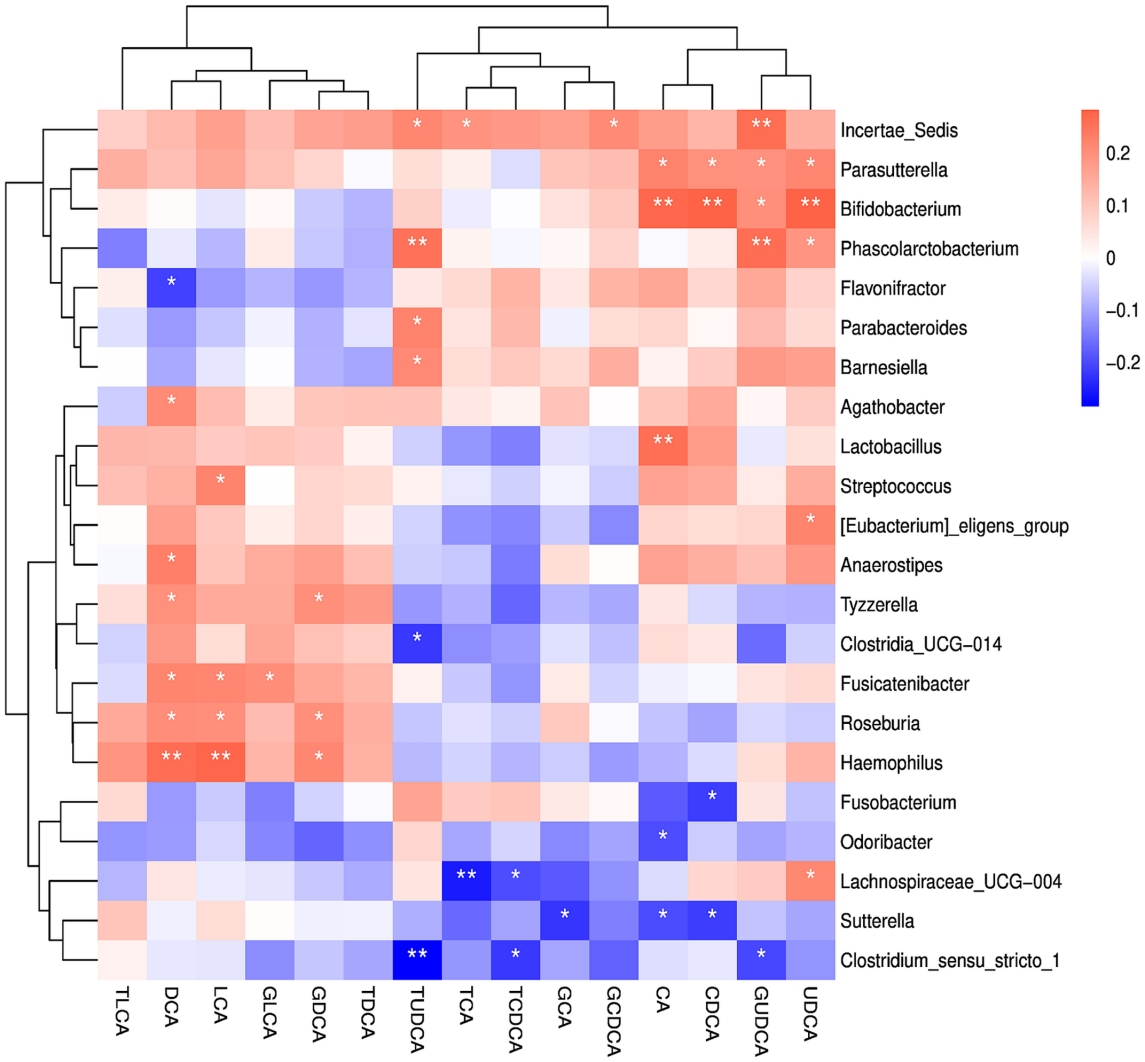
Correlation heatmap of bile acids and differential genus taxa. *Haemophilus*, *Fusicatenibacter*, and *Roseburia* showed positive correlations with both DCA and LCA. *Flavonifractor* showed negative correlation with DCA. *p < 0.05, **p < 0.01.

To investigate functional crosstalk, we analyzed correlations between genus-level taxa and bile acids. Our analysis identified putative co-metabolizers of secondary bile acids, with *Haemophilus*, *Fusicatenibacter*, and *Roseburia* showing positive correlations with both deoxycholic acid (DCA) and lithocholic acid (LCA) (Figure 7). Conversely, the negative correlation between DCA and *Flavonifractor* suggests a potential inhibitory relationship.

## 4 Discussion

The exact etiology of inflammatory bowel disease (IBD) remains unclear; however, it is considered to result from a complex interplay between host factors and microorganisms, including an imbalance in microbial homeostasis, a damaged intestinal mucosal barrier, and abnormal immune responses (Qiu et al., 2022). Generally, the disease burden of ulcerative colitis (UC) is believed to be lower than that of Crohn’s disease (CD), and consistent with this, the degree of gut microbiota dysbiosis in UC patients is also considered less severe than in CD patients (Qiu et al., 2022; Le Berre et al., 2020; Kriss et al., 2018). Our findings strongly support this notion, revealing that the gut microbiota disorder in CD patients is more severe, a conclusion drawn from the more pronounced enrichment of pro-inflammatory taxa and a profound, specific depletion of beneficial bacterial consortia.

A key differentiator in CD was the marked enrichment of pro-inflammatory genera. *Escherichia-Shigella* and *Ruminococcus gnavus* group were two main enriched genera in our IBD patients, which aligns with previous reports (Kim et al., 2024; Wang et al., 2024; Hall et al., 2017; Volkmann, 2023). The pathogenic potential of *Ruminococcus gnavus* is particularly notable, as it secretes a complex glucorhamnan polysaccharide that can induce dendritic cells to release TNF-*α* in a Toll-like receptor 4-dependent manner, thereby exacerbating intestinal inflammation (Henke et al., 2021; Henke et al., 2019). This provides a mechanistic basis for the more inflammatory environment we observed in CD.

The most striking finding was the extensive and specific depletion of short-chain fatty acid (SCFA)-producing bacteria exclusively in CD. We identified a consortium of seven genera, *Blautia*, *Lachnospira*, *Roseburia*, *Anaerostipes*, *Eubacterium eligens group*, *Romboutsia*, and *Butyricicoccus,* that were uniquely depleted in the CD group. Importantly, these seven genera are all reported SCFA-producing organisms (Zhou et al., 2022; Valencia-Buitrago et al., 2025; Esmail et al., 2025; Khassenbekova et al., 2025; Ilie et al., 2025) and predominantly belong to the *Lachnospiraceae* family. In contrast, UC exhibited a different pattern, with only *Odoribacter*, a butyrate producer (Aguirre-Ramírez et al., 2025), being specifically depleted in UC. As SCFAs like butyrate provide energy for intestinal epithelial cells, enhance barrier integrity, and regulate immune cell function (Deleu et al., 2021), the collective loss of these key producers in CD offers a compelling explanation for its more severe pathophysiology from a gut microbiota perspective.

This microbial dysbiosis was further reflected in host metabolism. We confirmed that secondary bile acids, including DCA, GDCA, TDCA, LCA and GLCA, were decreased in both UC and CD groups, consistent with recent serum-based findings (Xu et al., 2024). Given that secondary bile acids derived from primary bile acids by specific gut bacteria, primarily from the Lachnospiraceae and Ruminococcaceae families (Sinha et al., 2020), we investigated the link between our microbial and metabolic data. Our correlation analysis revealed that several Lachnospiraceae genera were positively correlated with DCA and LCA, including *Fusicatenibacter*, *Roseburia*, *Eubacterium eligens group, Agathobacter*, and *Anaerostipes*. This functionally links the depletion of these specific bacteria in CD to the observed deficiency in immunomodulatory secondary bile acids, whose role in maintaining gut immune homeostasis has been established (Cai et al., 2022; Lloyd-Price et al., 2019; Franzosa et al., 2019; Sinha et al., 2020).

An unexpected observation was the enrichment of *Bifidobacterium*, contrary to reports of its depletion and anti-inflammatory role in IBD (Ahmadi et al., 2025; Vallejos et al., 2025; Ni et al., 2023). This discrepancy may be attributed to some patients having received treatment with oral probiotics containing *Bifidobacterium* prior to sampling.

Several limitations should be acknowledged. First, the use of healthy first-degree relatives (HFDRs) of patients as a control group should be considered in further study (Chen et al., 2024). Second, the cross-sectional design precludes causal inference regarding the observed microbial–metabolic alterations. Third, the relatively modest sample size, while comparable to many published IBD microbiome studies, subsequent validation can be conducted in larger sample sizes. Fourth, the use of 16S rDNA sequencing restricts taxonomic resolution to genus level and provides only indirect inference of functional capacity; future studies employing shotgun metagenomics or metatranscriptomics would enable strain-level characterization and direct assessment of microbial gene expression. Fifth, potential confounding by medication use (e.g., antibiotics, 5-ASA, immunosuppressants, biologics) and dietary habits was not fully controlled, and future studies should incorporate rigorous covariate adjustment or prospective dietary intervention designs.

## 5 Conclusion

In summary, this integrated analysis demonstrates that CD is characterized by a more severe synergistic dysbiosis than UC, defined by a collapse of SCFA-producing Lachnospiraceae and expansion of pro-inflammatory pathobionts, which correlates directly with a deficit in protective secondary bile acids. These findings underscore the critical role of the Lachnospiraceae family in IBD pathophysiology and pinpoint its functional output as a key target for future microbiota-based therapeutic strategies.

## Data Availability

The original contributions presented in the study are included in the article/Supplementary material, further inquiries can be directed to the corresponding authors. The data presented in the study are deposited in the NCBI repository and MetaboLights repository, accession number PRJNA1437910 and MTBLS14084, respectively.
