# Supplementary material for: Gut microbiota and bile acids profiles study of ulcerative colitis and Crohn’s disease patients
Source: Front Microbiol. 2026 Apr 7;17:1782415. doi: 10.3389/fmicb.2026.1782415 (PMC13095740; doi:10.3389/fmicb.2026.1782415)
Supplement: Supplementary file 1 [file Supplementary_file_1.docx]

**Supporting information**


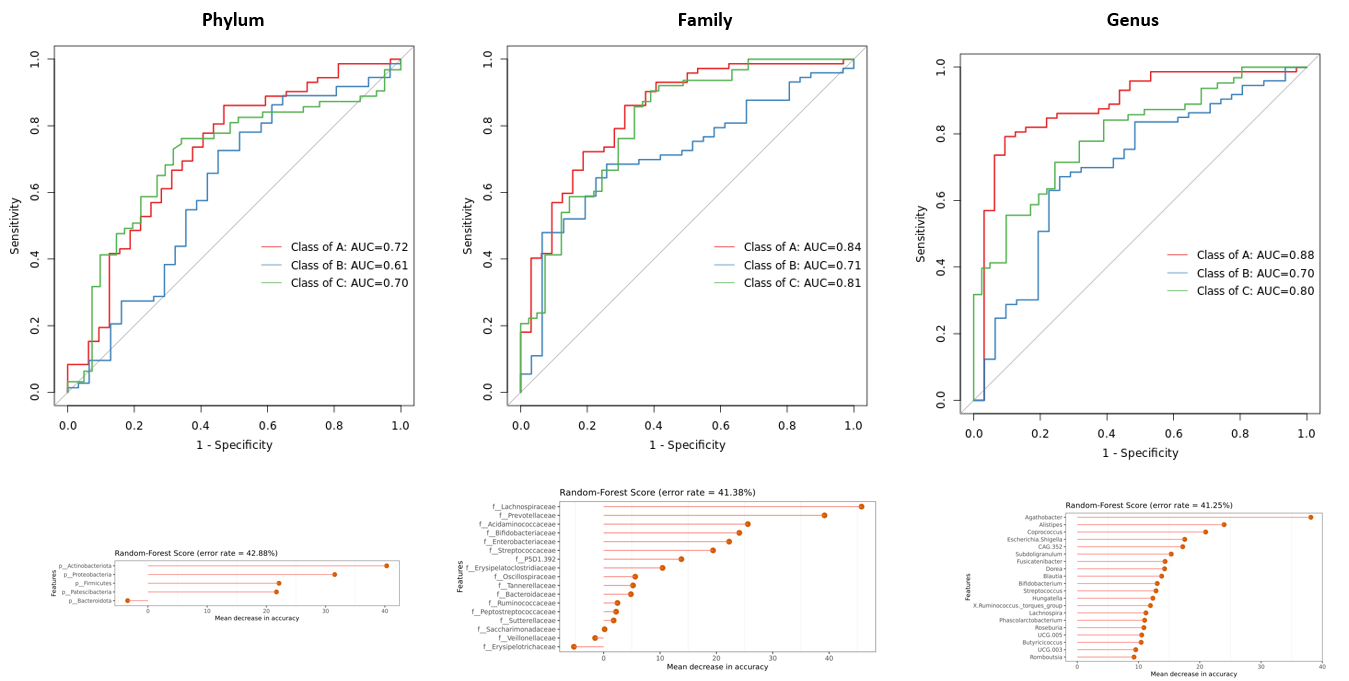


**Figure S1. ROC curve and random-forest score of IBD in phylum, family and genus level.**


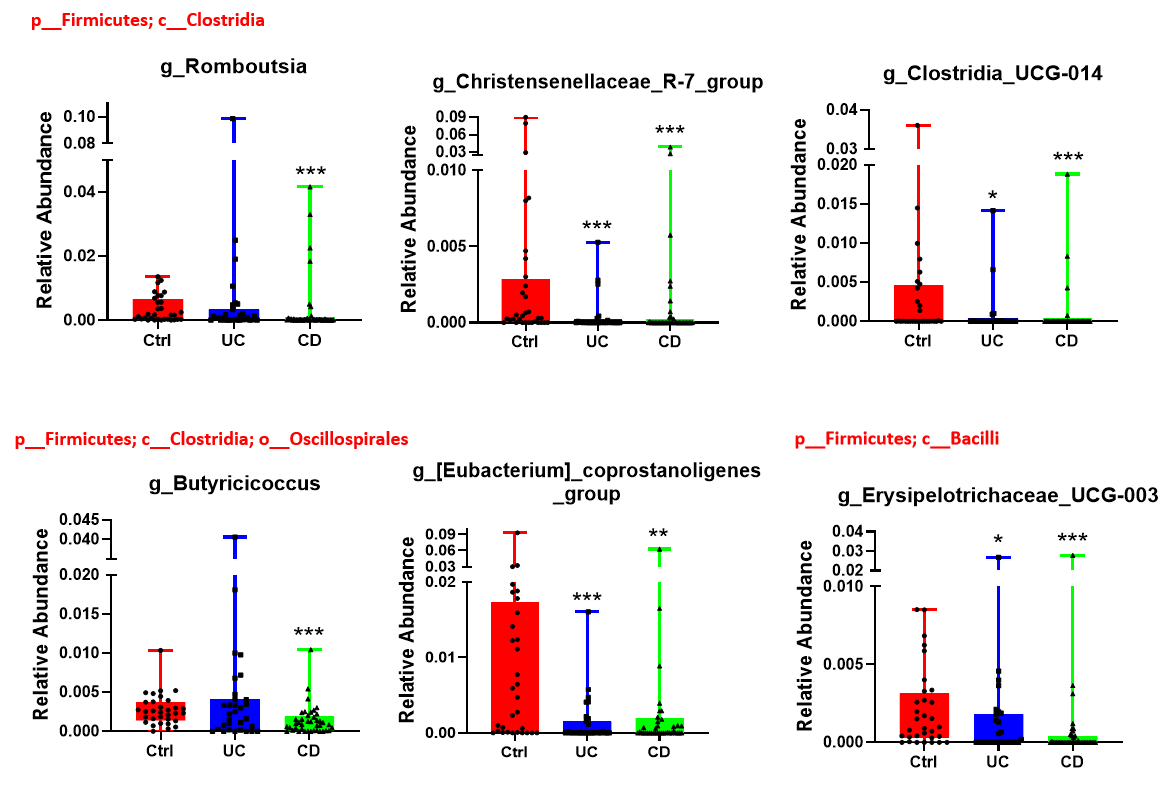


**Figure S2. Differential genus taxa of p_Firmicutes.**


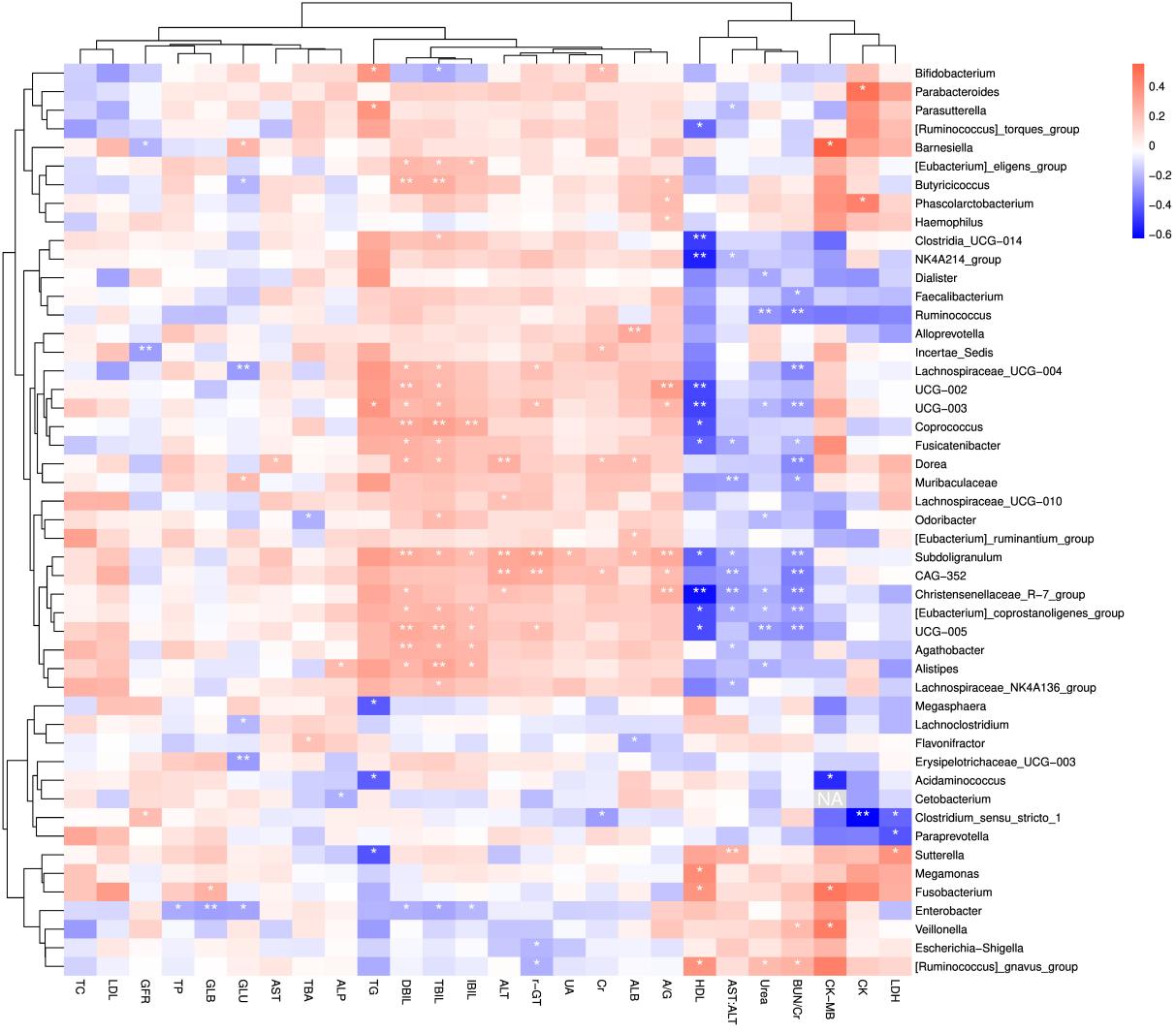


**Figure S3. Correlation heatmap of laboratory biochemical indicators and differential genus taxa.**
